# Verification of Medorrhinum

**Published:** 1892-12

**Authors:** J. H. Allen

**Affiliations:** Logansport, Ind.


### VERIFICATIONS OF MEDORRHINUM.

J. H. Allen, M. D., Logansport, Ind.

*Mind.*—Forgetful, cannot remember the least thing any length of time; writes everything down of any importance; cannot trust herself to remember it; great irritability and disgust with life.

*Head.*—Severe pain at the base of the brain, running to the vertex, in two cases suffering from retroflexion.

*Nose.*—Nose stopped up, cannot breathe through it in the morning; snuffles in children not relieved by other remedies.

*Urinary Organs.*—Frequent urination with slight burning at the meatus; dull heavy ache in the region of the prostate, marked in two cases; very sore in perineum extending to rectum, frænum red and swollen in three cases. Red vesicles on the glans penis that have a burning itching sensation, very irritable. Four cases cured.

Female urinary organs:

Urinates every half-hour; urgent, can't wait a moment, worse at or during the menstrual period.

Ovaries:

Sharp shooting pains like knives, much soreness and tenderness to pressure—the slightest pressure causes her to suffer pains for hours, bearing-down sensation, especially in the left ovary, relieved by pressing up on abdomen (like Lill.); pains run from left ovary to uterus.

*Uterus.*—Subinvoluted, sensitive to slightest pressure, a sensitive spot above and to the right of the cervix in six cases.

*Leucorrhœa.*—Thin, acrid, producing severe pruritis, itching intolerable, worse by rubbing (like Coffea), also better by bathing frequently with tepid water; it has odor of decayed fish; sharp pains run from uterus to rectum.

*Rectum.*—Sycosis of the rectum, seven cases, three in women and four in men, thin ichorous discharge from rectum, producing very intense itching and burning, which prevents sleep, worse the first part of the night, one case in a man had to use Opium suppositories in order to sleep. The rectal discharge in one case produced blisters on healthy flesh where it touched; there is also a full, stuffed feeling in the rectum.

*Throat and Lungs.*—Takes cold at the slightest exposure, begins in the head and goes down on the lungs; severe burning in the base of the tongue, extending down the bronchii as if he had inhaled hot steam; worse in the morning, a raw feeling extending from throat to lungs as if the mucous membrane was scraped with a knife; aggravated by breathing cold air, sensation as if the lungs were stuffed with cotton. Expectoration greenish yellow, catarrhal cold of the head with burning of septum of the nose to bronchii, complete loss of taste and smell —cannot taste tobacco.

*Gonorrhœal Rheumatism.*—Of the wrists and ankles with complete loss of power in the part affected. If in the ankle, was unable to walk; if in the wrist, it had to be carried in a sling.

DISCUSSION.

Dr. Tompkins—In how many of those cases did you suspect a history of gonorrhœa?

Dr. J. H. Allen—In all of them.

---

NOTE.—An excellent proving of Medorrhinum may be found in Hering's *Guiding Symptoms*, Vol. VII, pages 292 to 324. The symptoms brought out by Dr. Allen in the foregoing paper are the more valuable because they confirm so many of the symptoms given in the proving. The nosodes must become our most valuable allies in the treatment of disease when they are thoroughly proved and the symptoms confirmed by clinical observation.—EDITOR.

